# Cloning and functional analysis of glutathione S-transferase gene *BxGST3* and *BxGST1* in *Bursaphelenchus xylophilus*

**DOI:** 10.3389/fpls.2026.1847982

**Published:** 2026-05-25

**Authors:** Bo Nian, Jian-ren Ye, Xiao-Qin Wu

**Affiliations:** College of Forestry, Nanjing Forestry University, Nanjing, China

**Keywords:** *B. xylophilus*, glutathione S-transferase (GSTs), *in situ* hybridization, rapid-amplification of cDNA ends (RACE), RNAi

## Abstract

**Introduction:**

Pine wilt disease (PWD), caused by the pine wood nematode *Bursaphelenchus xylophilus*, is a devastating disease affecting pine forests worldwide. Glutathione S-transferases (GSTs) are key detoxification enzymes that protect organisms against oxidative stress and xenobiotic compounds. However, the roles of specific GST genes in *B. xylophilus* pathogenicity remain poorly understood.

**Methods:**

In this study, we cloned the full-length cDNAs of two GST genes, *BxGST3* and *BxGST1*, using rapid amplification of cDNA ends (RACE). Quantitative PCR was performed to examine their expression during infection of *Pinus thunbergii*. In situ hybridization was used to localize their expression in nematode tissues. RNA interference (RNAi) was employed to silence these genes and assess their effects on nematode physiology.

**Results:**

Both genes were up-regulated during infection, with peak expression at 24 hours post-inoculation (5.1-fold for *BxGST3* and 3.1-fold for *BxGST1*). *In situ* hybridization localized their expression to the esophageal glands, gonads, and intestine. RNAi-mediated silencing of *BxGST3* and *BxGST1* reduced feeding activity and reproduction, and delayed pine wilt symptoms.

**Discussion:**

These findings demonstrate that *BxGST3* and *BxGST1* contribute to *B. xylophilus* pathogenicity, likely through detoxification of host defense compounds.

## 1 Introduction

Pine Wilt Disease is a typical pathogen-driven disease. Pine wood nematode (PWN) causes extensive mortality in pine forests wherever it occurs. The disease originated in North America (Zhang et al., 2020). Its primary mode of transmission involves the Japanese pine sawyer beetle (*Monochamus alternatus*) (Li et al., 2022), which carries the nematode and introduces it into pine trees during feeding and oviposition, leading to successful infection. Once infected, pine trees can wilt and die within 60–90 days, with some cases succumbing in just over 40 days. The disease spreads rapidly and is often described as the “cancer of pine trees” (Xiao et al., 2024; Ye, 2010). This pathogen poses a serious threat to forest ecosystems and has resulted in substantial economic losses. In 2017 alone, pine wilt disease in mainland China affected 85,524 hectares of pine forest, causing an estimated economic loss of approximately 19.5 billion RMB, including 3.5 billion RMB in direct losses and 16 billion RMB in indirect losses (Zhang et al., 2020). In addition to its impact on flora and fauna, pine wilt disease also adversely affects the human environment (Kim et al., 2020).

In recent years, Pine wilt disease prevention and control is now a central focus in the field, and gene-targeted approaches are increasingly seen as a promising research avenue. With the progress of molecular biology, much of the spotlight has fallen on functional studies of genes linked to *B. xylophilus*—especially those governing its growth, development, and pathogenicity (Liu et al., 2025; Wang et al., 2025)—as well as genes involved in the active defense of pine trees (Xie et al., 2023; Xu et al., 2026). Glutathione S-transferases (GSTs) are a group of multifunctional isozymes widely distributed in mammals, plants, insects, parasites, and microorganisms. GSTs are recognized as key players in phase II detoxification, catalyzing the conjugation of glutathione to xenobiotic compounds and protecting organisms from oxidative stress. Many genes in the *GST* family have been confirmed through years of research to participate in detoxification mechanisms in organisms (Cui et al., 2020) and protect them from various stressors (Muslu et al., 2024; Kim et al., 2016; Dancy et al., 2016; Hasegawa et al., 2010). In recent years, RNA interference (RNAi) technology has been increasingly used to investigate the functions and characteristics of genes related to *B. xylophilus*. For example, studies on genes such as *Bx-CYP* (Xu et al., 2015), *Bx-ATG* (Deng et al., 2016), and *Bx-srh* (Cao et al., 2023) have confirmed their strong association with the pathogenicity of the nematode. In parasitic nematodes, GSTs have been shown to play crucial roles in stress resistance and represent potential genetic targets for control (Fu et al., 2020; Yang et al., 2021; Zhou et al., 2020). Although GSTs have been studied in *B. xylophilus*, the specific functions of *BxGST3* and *BxGST1*—including their expression dynamics during host infection, tissue localization, and contributions to feeding, reproduction, and pathogenicity—remain unknown. Unlike *GST* genes from other parasitic lineages, which participate in processes such as anti-aging (Gao et al., 2025; Gramberg et al., 2024) and arsenic detoxification (Kaiser et al., 2025), *BxGST3* and *BxGST1* may fulfill different physiological functions.

We hypothesized that (1) *BxGST3* and *BxGST1* are up-regulated during host infection to support detoxification; (2) these genes are expressed in tissues involved in stress response and reproduction; and (3) silencing these genes would reduce nematode fitness and pathogenicity. This study aims to obtain the full-length sequences of two *GST* genes, *BxGST3* and *BxGST1*, from *B. xylophilus* using RACE technology. It further explores changes in the expression levels of these genes during the nematode-plant interaction and conducts *in situ* hybridization for localization analysis. Additionally, RNA interference was employed to examine the effects of these two genes on a range of physiological characteristics of *B. xylophilus*. The findings are expected to provide a scientific basis for future research on the pathogenic mechanisms of *B. xylophilus* and to offer new strategies and theoretical foundations for more effective control of pine wilt disease.

## 2 Results

### 2.1 Full-length cDNA amplification and sequence analysis of the *BxGST3* and *BxGST1* genes from *B. xylophilus*

**Figure 1:**
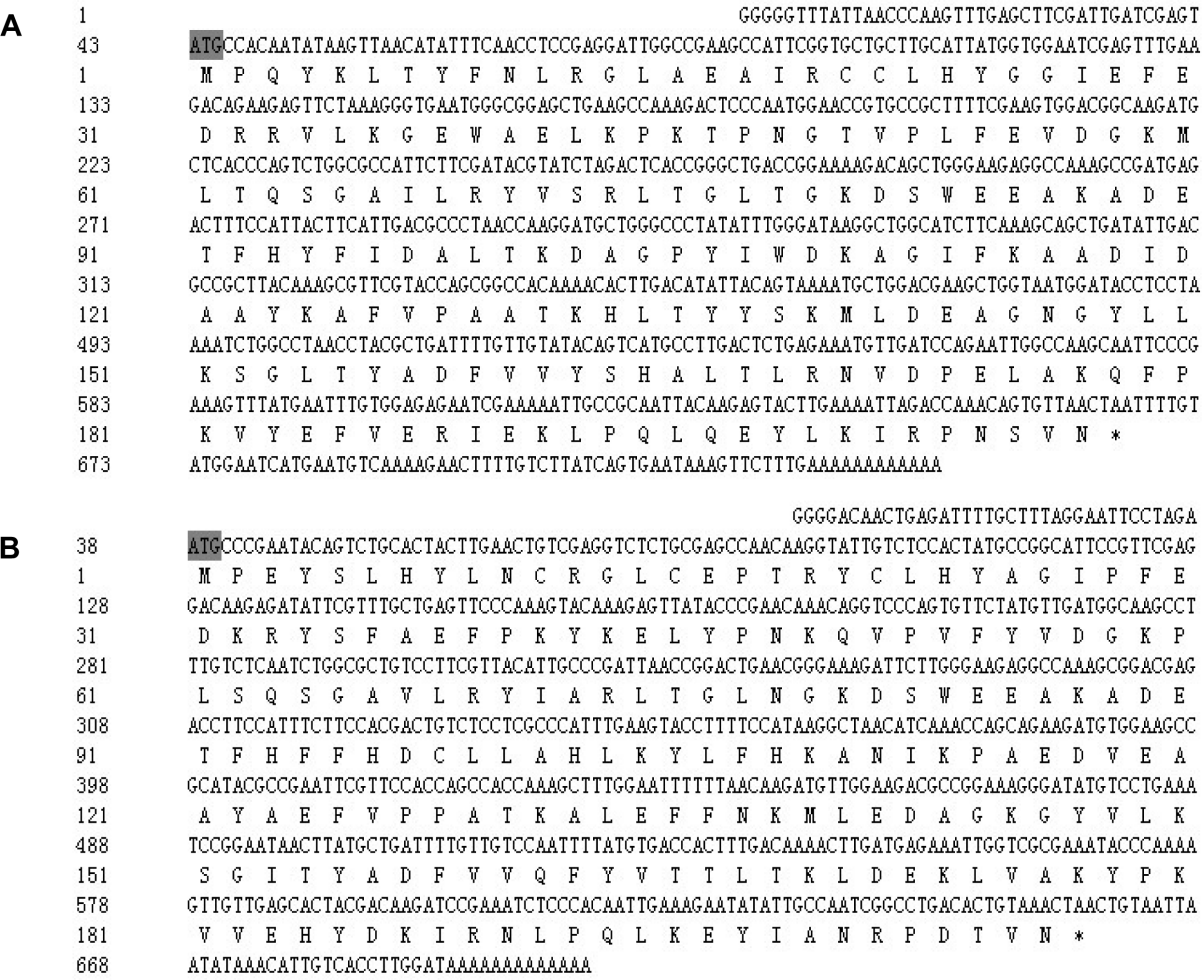
Full-length cDNA sequences and deduced amino acid sequence of *BxGST3* **(A)** and *BxGST1* **(B)** from *B. xylophilus*.

The full-length sequences of the *BxGST3* and *BxGST1* genes were obtained by RACE amplification (**Figure 1**). Analysis using DNAMAN software showed that the *BxGST3* cDNA from *B. xylophilus* is 738 bp in length, encoding a protein of 207 amino acids. The coding sequence (CDS) of *BxGST3* is flanked by a 5’ untranslated region (UTR) spanning nucleotides 1–41 bp and a 3’ UTR spanning nucleotides 666–738 bp, with the CDS located between nucleotides 42 and 665 bp of the full-length cDNA. The *BxGST1* cDNA is 702 bp in total length, encoding 206 amino acids. Its CDS is flanked by a 5’ UTR spanning nucleotides 1–36 bp and a 3’ UTR spanning nucleotides 658–702 bp, with the CDS located between nucleotides 37 and 657 bp. (The nucleotide composition analysis has been removed as suggested by the reviewer.)Further analysis using the Compute pI/Mw tool predicted the following physicochemical properties for the deduced proteins: the theoretical isoelectric point (pI) of *BxGST3* is 6.91, its molecular weight is 23.56 kDa. For *BxGST1*, the predicted pI is 7.04, its molecular weight is 23.87 kDa.

### 2.2 Alignment of the two predicted amino acid sequences

**Figure 2:**
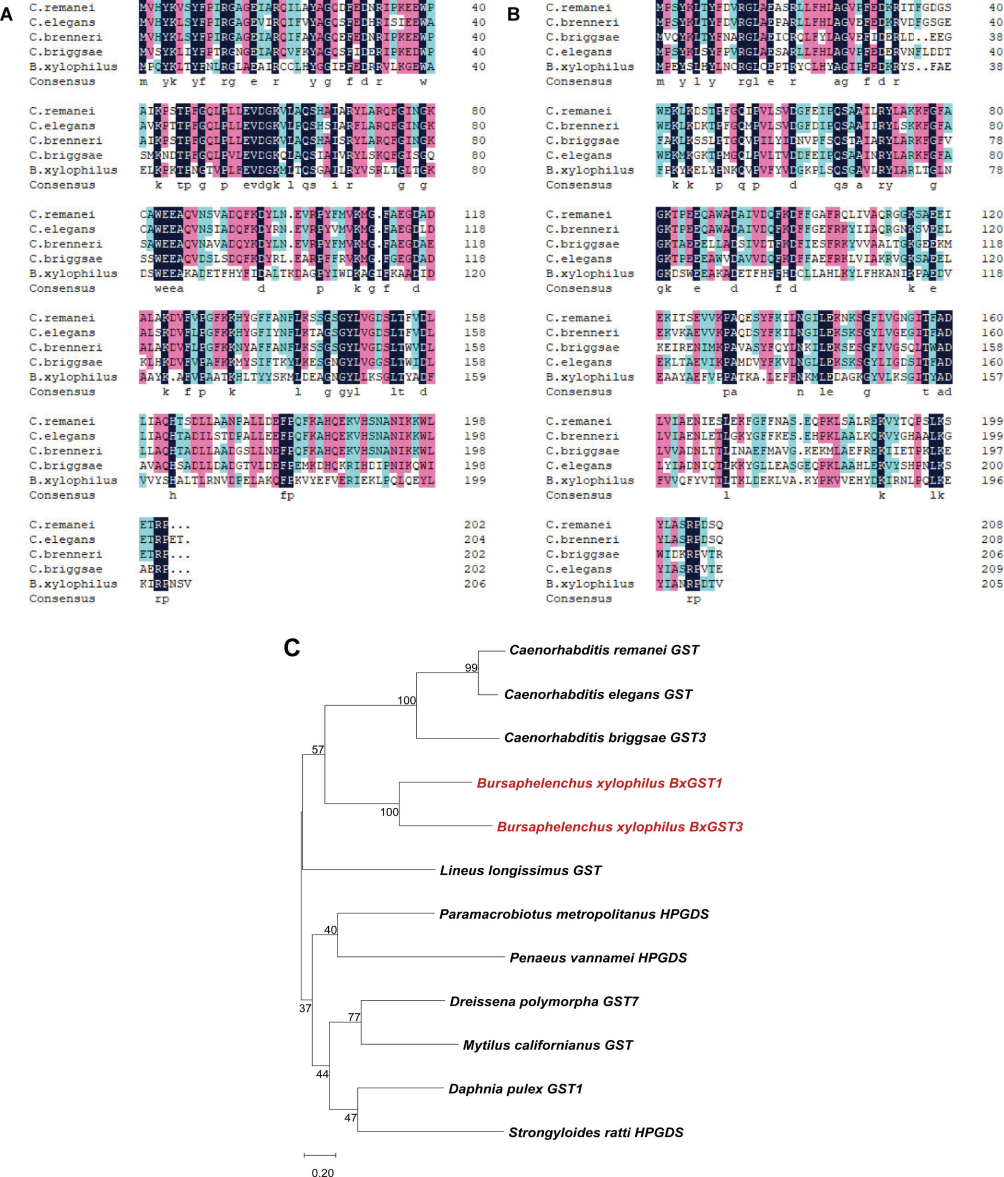
Comparison for protein homology of *BxGST3* **(A)** and *BxGST1* **(B)** from *C. elegans*, *C. remanei*, *C. briggsae*, *C. brenneri*, *B. xylophilus*. **(C)** Phylogenetic analysis of *BxGST3* and *BxGST1* with other GST, glutathione S-transferase protein sequences. The ML, maximum likelihood phylogenetic tree was constructed using MEGA12 software based on the full-length amino acid sequences of GSTs from *B. xylophilus* and other representative species. The sequences used in this analysis are as follows: XP_064633951.1 (*Lineus longissimus*), XP_003108807.2 (*Caenorhabditis remanei*), XP_052232067.1 (*Dreissena polymorpha*), XP_052067745.1 (*Mytilus californianus*), XP_055328745.1 (*Paramacrobiotus metropolitanus*), NP_497119.1 (*C. elegans*), XP_002633726.1 (*C. briggsae*), XP_069999240.1 (*Penaeus vannamei*), XP_046447488.1 (*Daphnia pulex*), and XP_024508723.1 (*Strongyloides ratti*). Bootstrap values (based on 1,000 replicates) are shown at the nodes. The scale bar =0.20.

Blastp alignment was performed using the predicted protein sequences. The results revealed that the amino acid sequence of *B. xylophilus BxGST3* exhibits high similarity to the *GST-3* protein sequences of *C. elegans*, *C. briggsae*, *C. remanei*, and *C. brenneri*, with sequence similarity ranging between 50% and 60%. Similarly, the amino acid sequence of *B. xylophilus BxGST1* shows high similarity to the *GST-1* protein sequences of the same *Caenorhabditis* species, with sequence similarity ranging between 40% and 50%. The amino acid sequences were downloaded locally, and multiple sequence alignment was conducted using DNAMAN software (**Figure 2A**). To further elucidate the evolutionary relationships of *BxGST3* and *BxGST1*, a phylogenetic tree was constructed using the maximum likelihood (ML) method in MEGA12 software based on the amino acid sequences of these two genes and GSTs from other representative species (**Figure 2B**). The results showed that both *BxGST3* and *BxGST1* clustered within the nematode clade. Specifically, *BxGST1* clustered with *GST-3* sequences from *Caenorhabditis* species (including *C. elegans*, *C. briggsae*, and *C. remanei*) with high bootstrap support (99–100%), indicating a close evolutionary relationship. *BxGST3* also grouped within this cluster, albeit with moderate bootstrap support (57%). In contrast, GST sequences from non-nematode species (including mollusks, crustaceans, and flatworms) formed a distinct outgroup clade. These phylogenetic results further support the classification of *BxGST3* and *BxGST1* as nematode-specific GSTs and suggest their functional conservation within the phylum Nematoda (**Figure 2C**).

### 2.3 qPCR analysis of two genes in *B. xylophilus* before and after pine inoculation

**Figure 3:**
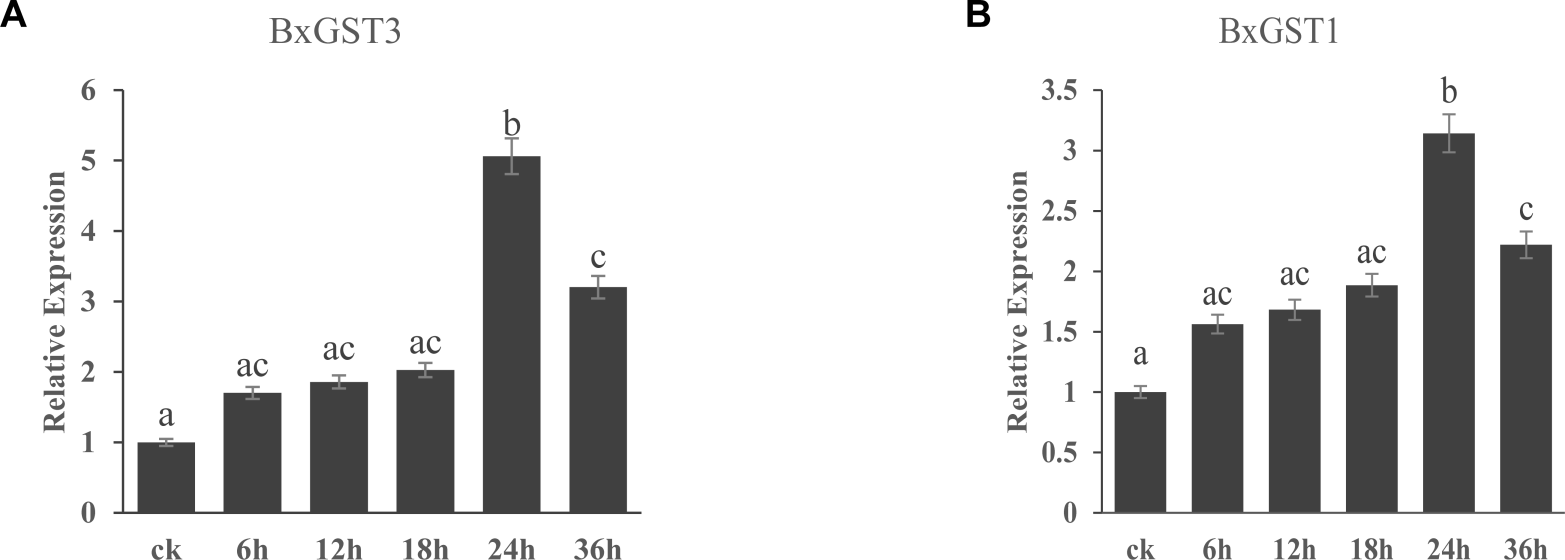
Analysis of *BxGST3* **(A)** and *BxGST1* **(B)** relative expression after *B. xylophilus* inoculation with *Pinus thunbergii* at different time points. Data are presented as mean ± SD (n = 3 biological replicates). Different letters indicate statistically significant differences (P < 0.05) as determined by one-way ANOVA followed by Duncan’s multiple range test.

qPCR analysis showed that the relative expression levels of the *BxGST3* and *BxGST1* genes in *B. xylophilus* increased from 6 to 36 hours after inoculation onto *Pinus thunbergii*. The expression rose gradually at 6, 12, and 18 hours, peaking at 24 hours with expression levels 5.063-fold and 3.143-fold higher for *BxGST3* and *BxGST1*, respectively. Significant up-regulation was observed at 24 h for both genes (*BxGST3*: 5.063-fold, P < 0.05; *BxGST1*: 3.143-fold, P < 0.05) compared to the 6 h time point. Subsequently, expression began to decline at 36 hours post-inoculation (**Figure 3**). These results demonstrate that *BxGST3* and *BxGST1* are up-regulated during the interaction between *B. xylophilus* and its pine host, with *BxGST3* showing a more pronounced response.

### 2.4 *In situ* hybridization

**Figure 4:**
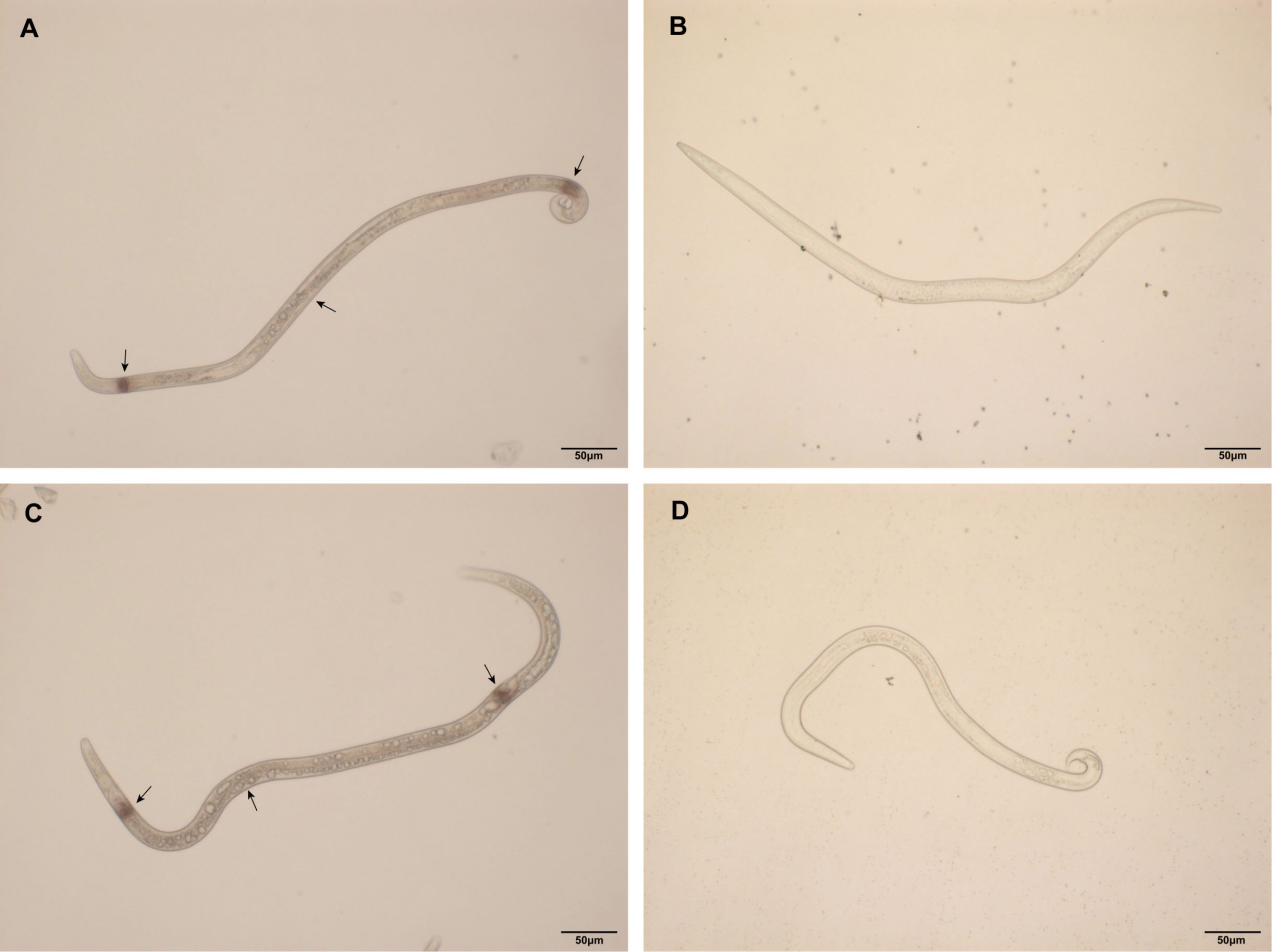
*In situ* hybridization result of *BxGST3* and *BxGST1* genes. **(A)** Hybridization signal of *BxGST3*; **(B)** Negative control (without probe); **(C)** Hybridization signal of *BxGST1*; **(D)** Negative control (without probe). Black arrows indicate positive signals in eg, esophageal glands and g, gonads. Scale bars = 50 μm.

Nematodes collected 24 hours after inoculation of *Pinus thunbergii* were processed for *in situ* hybridization and subsequently observed and photographed under a Zeiss upright microscope (Zeiss Axio Imager M2, Germany) (**Figure 4**). In the control group, where nematodes were processed without probe hybridization, no specific labeling signal was detected on the nematode body. *In situ* hybridization revealed strong brown-black staining in the esophageal glands and gonads of *B. xylophilus*, with weaker staining in the intestine (**Figures 4A, C**). No staining was observed in the negative control (**Figures 4B, D**). These results indicate that both *BxGST3* and *BxGST1* are primarily expressed in the esophageal glands and gonads, with secondary expression in the intestine.

### 2.5 RNAi efficiency and phenotypic analysis

**Figure 5:**
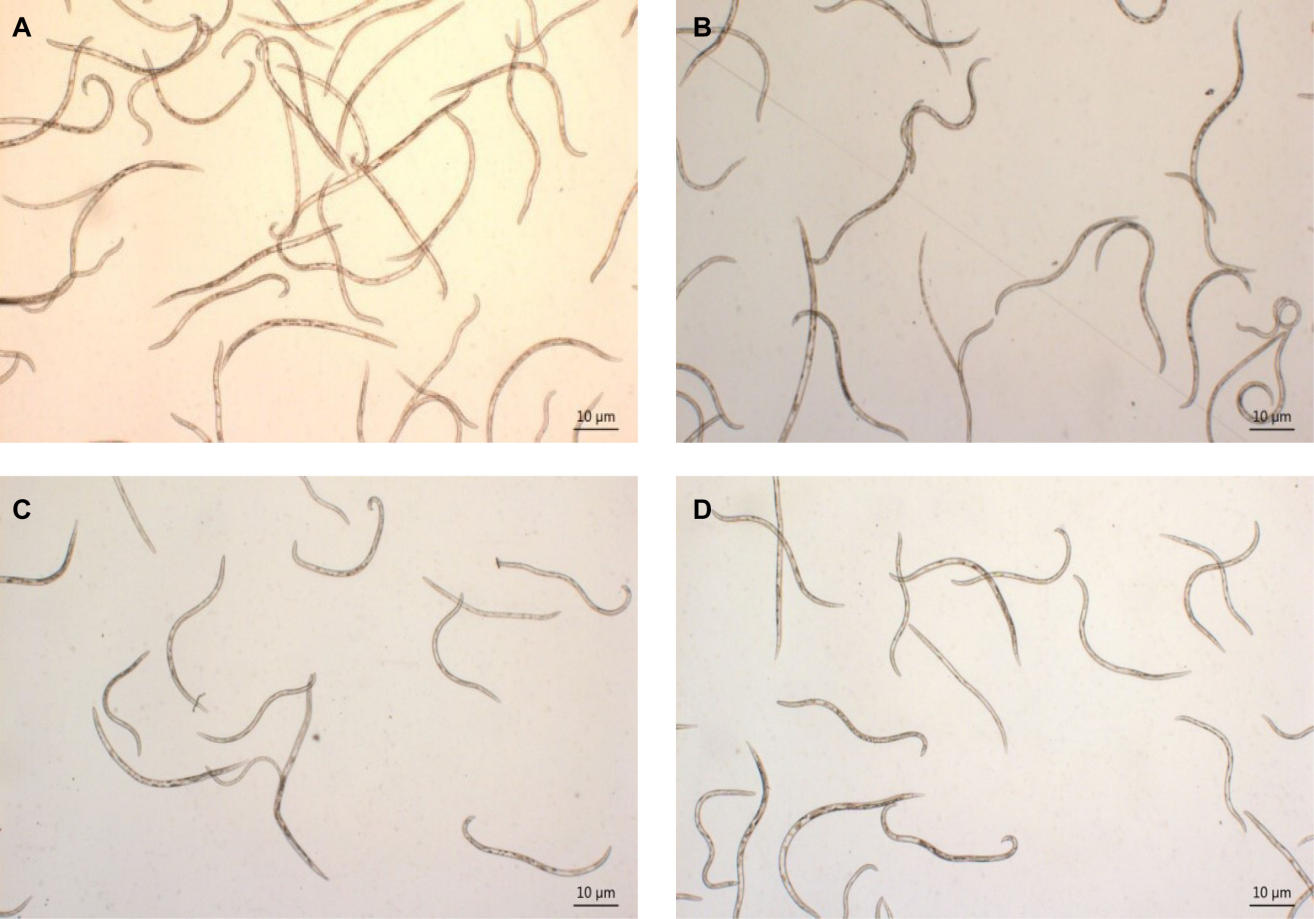
Morphological observation of *B. xylophilus* following RNAi treatment. Scale bars = 10 μm. Nematodes were soaked in sterile water **(A)**, ds*GFP* **(B)**, ds*BxGST3* **(C)**, ds*BxGST1* **(D)** for 48 h.

**Figure 6:**
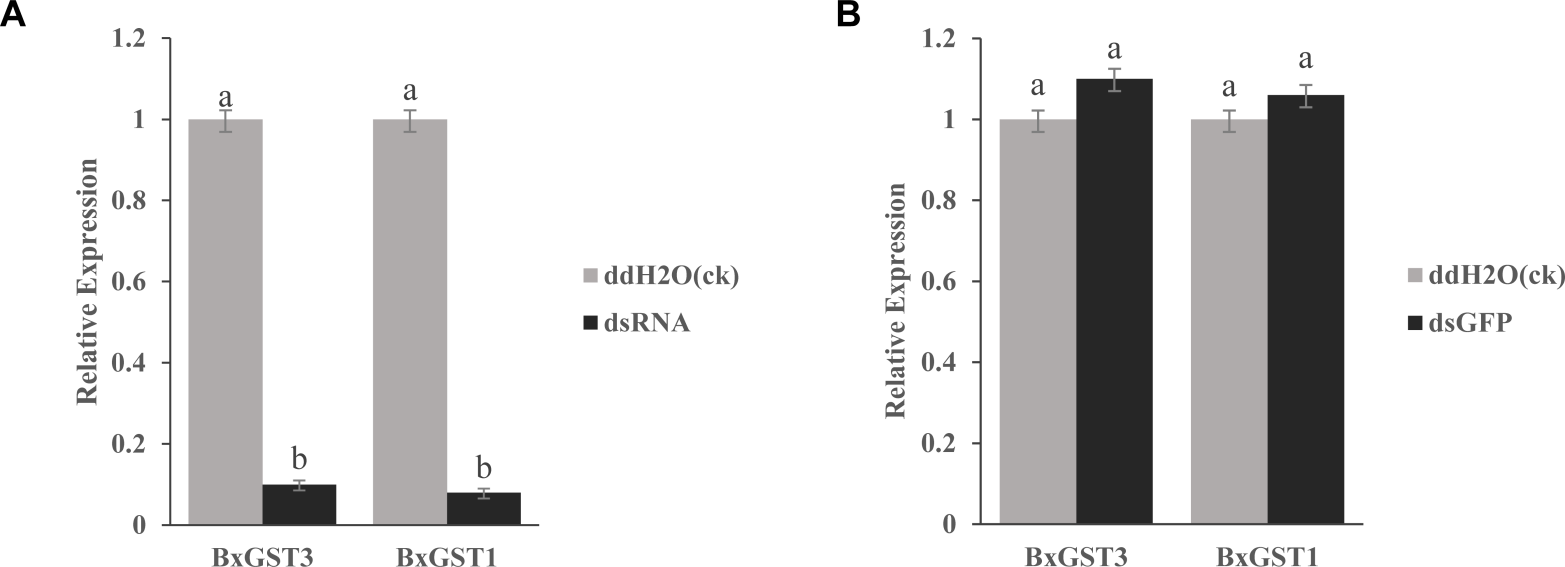
*BxGST3* and *BxGST1* expression after dsRNA treatment **(A)**; Expression after ds*GFP* treatment **(B)**. Data are presented as mean ± SD (n = 3). Different letters indicate statistically significant differences (P < 0.05) as determined by Duncan's multiple range test.

**Figure 7:**
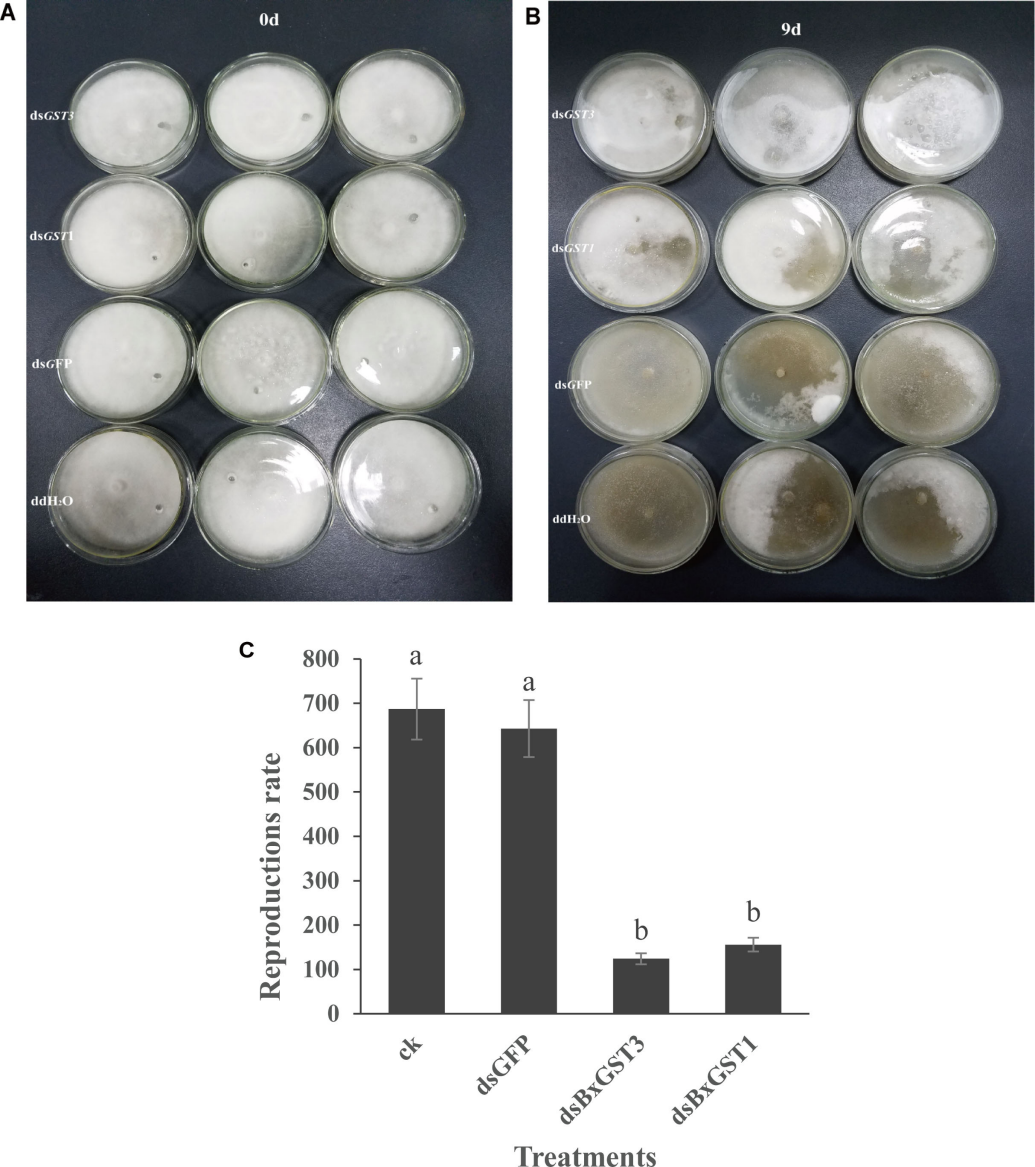
Feeding and reproductive capacity of *B. xylophilus* on *Botrytis cinerea* plates following RNAi. **(A)** Feeding activity at day 0; **(B)** Feeding activity at day 9; **(C)** Reproduction multiplier. Data are presented as mean ± SD (n = 3). Different letters indicate statistically significant differences (P < 0.05) as determined by one-way ANOVA followed by Duncan's multiple range test.

In this experiment, the morphology of PWNs was observed after 48 h of soaking in ds*BxGST3*, ds*BxGST1*, ds*GFP* solutions, or sterile water (**Figure 5**). The results showed that the treated nematodes exhibited no obvious differences in size or morphology compared with the control group, and no significant mortality was observed. To evaluate interference efficiency, real-time qPCR was performed to measure changes in mRNA expression after dsRNA treatment. The results showed that the expression of *BxGST3* decreased by 90% compared with the control group, while that of *BxGST1* decreased by 92% (**Figure 6A**). In contrast, no significant difference in expression of either gene was observed between nematodes soaked in ds*GFP* interference solution and those soaked in sterile water (**Figure 6B**). These findings indicate that treatment with ds*BxGST3* and ds*BxGST1* effectively suppressed the mRNA expression of the corresponding target genes. As shown in **Figure 7B**, after 9 days, the feeding activity of PWNs treated with ds*BxGST3* or ds*BxGST1* showed little change compared to that at day 0 (**Figure 7A**), producing feeding zones only 1–3 cm in diameter. In contrast, nematodes treated with ds*GFP* or sterile water formed significantly larger feeding zones of 7–10 cm in diameter. After 9 days of reproduction, the reproduction multiplier for nematodes treated with sterile water reached 687-fold, while those treated with ds*GFP*, ds*BxGST3*, and ds*BxGST1* showed reproduction multipliers of 643-, 124-, and 156-fold, respectively (**Figure 7C**). This indicates that silencing the glutathione S-transferase genes *BxGST3* and *BxGST1* compromised the reproductive capacity of the nematodes.

### 2.6 Pathogenicity of *B. xylophilus* after RNAi

**Figure 8:**
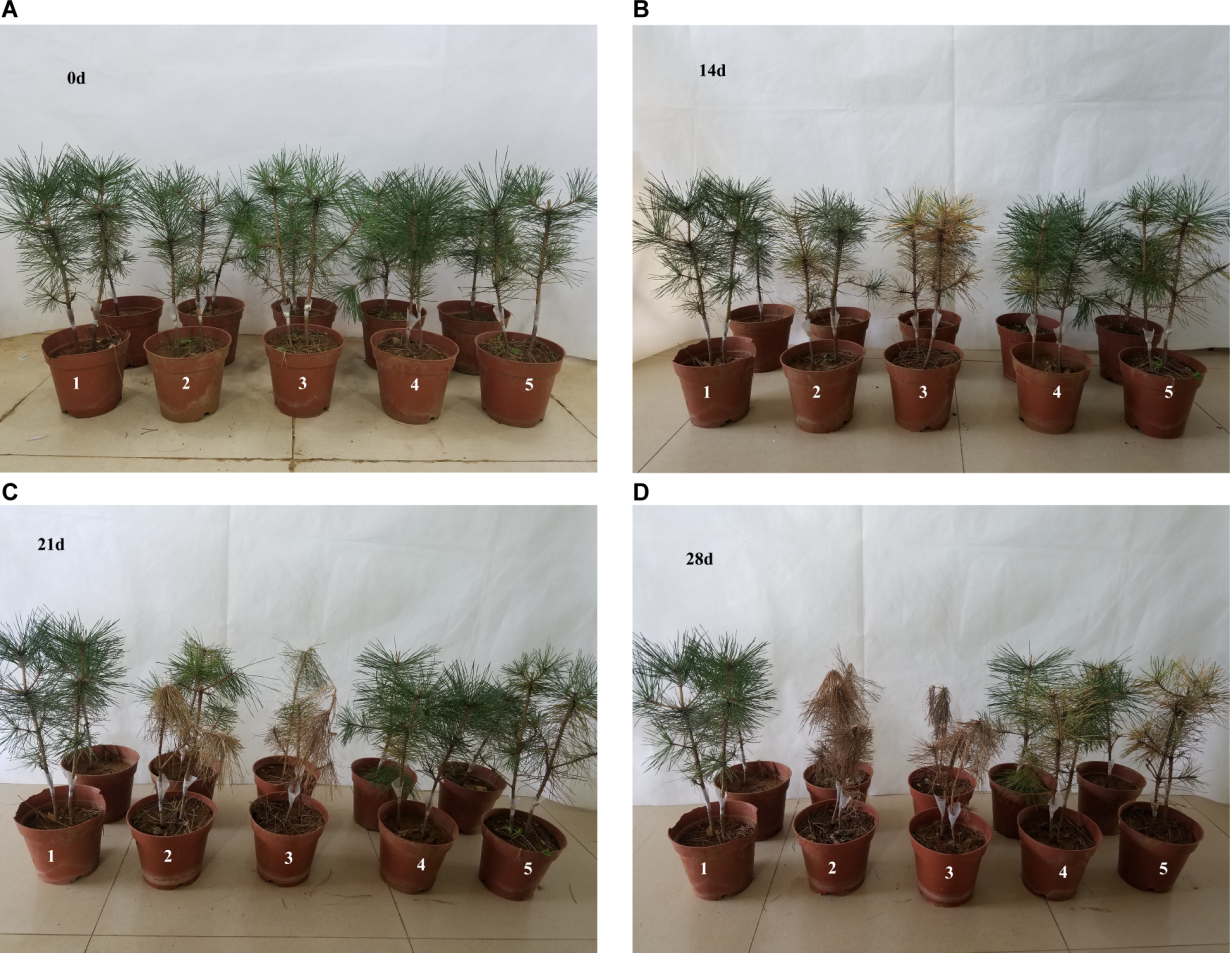
Symptoms in *P. thunbergii* seedlings 0 **(A)**, 14 **(B)**, 21 **(C)**, 28 **(D)** days after inoculation with Nematodes subjected to different treatments. Line 1: inoculated with sterile water; Line 2: inoculated with nematodes soaked in sterile water; Line 3: inoculated with nematodes soaked in ds*GFP* interference solution; Line 4: inoculated with nematodes soaked in ds*BxGST3* interference solution; Line 5: inoculated with nematodes soaked in ds*BxGST1* interference solution.

Nematodes subjected to different treatments were inoculated onto two−year−old *Pinus thunbergii* seedlings. Disease progression in the pine seedlings was monitored regularly to evaluate the impact of silencing the glutathione S−transferase genes *BxGST3* and *BxGST1* on the pathogenicity of *B. xylophilus* (**Figure 8**). Regular observations showed that seedlings inoculated with nematodes soaked in sterile water or ds*GFP* interference solution began to develop a small number of yellowish−green needles at 10 days post−inoculation (dpi), followed by a large number of such needles at 14 dpi, although the needles did not curl. In contrast, seedlings inoculated with nematodes treated with ds*BxGST3* or ds*BxGST1* displayed only a few diseased needles around 14 dpi. By 21 dpi, most needles on seedlings inoculated with nematodes soaked in sterile water or ds*GFP* had turned brown and died, and the shoot tips drooped. Seedlings inoculated with ds*BxGST3*− or ds*BxGST1*−treated nematodes merely showed more diseased needles than at 14 dpi, with no drooping of shoot tips. At 28 dpi, seedlings inoculated with sterile−water− or ds*GFP*−treated nematodes had completely browned and died, with the whole plant wilted and drooping. Seedlings inoculated with ds*BxGST3*− or ds*BxGST1*−treated nematodes began to exhibit extensive needle yellowing and loss of green color at 32 dpi, though the needles were not yet wilted or drooping. Subsequent observations revealed that seedlings inoculated with ds*BxGST3*− and ds*BxGST1*−treated nematodes completely wilted at 45 dpi and 42 dpi, respectively. These results demonstrate that interference with the *BxGST3* and *BxGST1* genes significantly reduces the pathogenicity of *B. xylophilus*.

## 3 Discussion

Pine wilt disease is widely considered the most destructive disease affecting pine trees, and it has drawn considerable attention from researchers around the world. Studies have shown that 60 genes, including GSTs, are up-regulated during the interaction between *B. xylophilus* and its plant hosts (Tsai et al., 2016). In addition, some research has confirmed that the expression levels of *Bx-GST* genes increase significantly when *B. xylophilus* is exposed to pesticides (Hao et al., 2023). A growing body of evidence indicates that GSTs play important roles in the physiological development of nematodes (Yang et al., 2021; Zhou et al., 2020). However, the specific functions of *BxGST3* and *BxGST1* in the physiology and pathogenicity of *B. xylophilus* have not been reported previously. While earlier work has characterized other GSTs in this species—such as *BxGST12* in pesticide detoxification (Hao et al., 2023)—this study is the first to describe the tissue-specific expression patterns and functional roles of *BxGST3* and *BxGST1* in feeding, reproduction, and host infection. In this study, we obtained the full-length cDNAs of *BxGST3* and *BxGST1* from *B. xylophilus* using RACE. Expression of both genes rose sharply after inoculation into pine hosts, peaking at 24 hours. *In situ* hybridization revealed that these two genes are expressed in the esophageal glands, gonads, and nearby intestinal regions of the nematode. A similar tissue distribution has been noted for *GST-p24* in *C. elegans* (Leiers et al., 2003) and for detoxification-related genes in *B. xylophilus* itself (Espada et al., 2016), indicating that the spatial segregation of detoxification functions—particularly between feeding-associated glands and digestive or reproductive tissues—may be a conserved feature across nematode species.

Our results suggest that silencing *BxGST3* and *BxGST1* reduces nematode fitness—specifically feeding and reproduction—which may in turn lower pathogenicity indirectly, rather than by directly eliminating virulence. We propose that *BxGST3* and *BxGST1* support feeding and reproduction by detoxifying host defense compounds that would otherwise damage the nematode’s digestive and reproductive tissues. When these GSTs are absent, accumulated toxins may impair esophageal gland function (leading to reduced feeding efficiency) and cause oxidative damage to gonadal tissues (leading to reduced fecundity). That said, we cannot rule out the possibility that these genes also have additional functions beyond detoxification. GSTs in other organisms have been shown to participate in cell signaling and stress response pathways that are not directly related to detoxification (Pajaud et al., 2012). Whether *BxGST3* and *BxGST1* play similar roles in *B. xylophilus* remains unclear. Our current data do not allow us to distinguish between a pure detoxification defect and broader effects on development or reproduction. This is a question worth pursuing in future studies, for example by examining whether the expression of other stress-related genes is altered after RNAi or by directly measuring toxin accumulation in nematode tissues. Similar to findings in *Trichinella spiralis* (Yang et al., 2021) and *Haemonchus contortus* (Zhou et al., 2020), *BxGST3* and *BxGST1* appear to support reproduction and stress tolerance. However, unlike the heme-regulatory role of Hc-hrg-2, our study provides the first evidence linking GST expression in the esophageal glands to feeding efficiency—a finding not previously reported in plant-parasitic nematodes.

Recent advances in single-cell and spatial transcriptomics have revealed that plant defense responses to pathogens are highly heterogeneous across cell types, a level of detail often lost in bulk RNA-seq analyzes (Ali et al., 2026). Complementing this, reactive oxygen and nitrogen species (ROS/RNS) play central roles in plant defense, with their biosynthesis and spatiotemporal signaling dynamics tightly regulating plant immunity and stress adaptation (Ali et al., 2025). Our tissue-specific localization of *BxGST3* and *BxGST1* to the nematode’s esophageal glands and gonads adds a pathogen-side perspective to these host-focused findings: detoxification functions are also spatially organized within the pathogen itself. When Ali et al. (2025) discuss how plants balance ROS/RNS production and scavenging to maintain optimal health, and Yu et al. (2012) show that NO and H_2_O_2_ levels rise significantly during pine–pine wood nematode interactions, our data suggest that nematodes may use GSTs in specific tissues to directly counter these host-generated oxidative stresses. This evidence from the nematode side—bridging host single-cell defense landscapes with pathogen tissue-specific detoxification—reinforces the idea that cell-type-resolved mechanisms on both sides shape the outcome of plant–nematode interactions.

Several limitations of this study should be noted. First, the pathogenicity assay used only two biological replicates because the experiment had already been completed. Nevertheless, the two replicates gave consistent results, and statistical analysis confirmed significant differences. Second, we do not yet know which host compounds *BxGST3* and *BxGST1* actually detoxify. Third, the RNAi effect was temporary, and off-target effects cannot be completely ruled out, although gene-specific fragments and the ds*GFP* control minimize this concern. For future work, we plan to include more biological replicates, measure GST enzyme activity and glutathione redox status after RNAi, test combined knockdown of both genes, examine expression in other pine hosts and nematode populations, identify upstream regulators, and identify the specific substrates of these two GSTs.

## 4 Materials and methods

### 4.1 Materials

The PWNs strain used in this study was AmA3 (Anhui province, China), and currently maintained in a university laboratory. The test pine seedlings were two-year-old *Pinus thunbergii* (approximately 30 cm in height) obtained from a greenhouse.

### 4.2 Nematode culture and host infection

*Botrytis cinerea* was cultured on potato dextrose agar (PDA) plates at a temperature of 25 °C ± 1 °C for 4–5 days in an incubator. After the PDA plates were fully covered with *B. cinerea* mycelia, the test *B. xylophilus* were introduced. Under the same conditions, the nematodes were allowed to feed on the fungus for 7–8 days until the mycelia were completely consumed. The nematodes were then collected using the Baermann funnel technique (Viglierchio and Schmitt, 1983). The harvested nematodes were counted under a microscope. Healthy two-year-old *Pinus thunbergii* seedlings were selected for artificial inoculation via the bark-inoculation method. Using a sterile blade, a cut was made through the bark into the xylem at approximately 10 cm above the ground on each seedling stem. A sterile cotton ball was inserted into the wound with forceps, and 5000 nematodes were inoculated into each seedling (Zhu et al., 2012). The inoculation start time was recorded. At different time points post-inoculation, the pine seedlings were cut at the base with pruning shears. After removing the needles, the stems were cut into small segments. Nematodes were collected from the segments using the Baermann funnel technique and immediately frozen in liquid nitrogen.

### 4.3 RNA extraction and quality assessment of *B. xylophilus*

After adding liquid nitrogen to the PWNs samples, they were thoroughly ground in a mortar. Total RNA of PWNs was then extracted using the Trizol method (Invitrogen, USA). RNA quality was assessed by 1% agarose gel electrophoresis (**Supplementary Figure 1**) and by OD260/280 ratio (1.8–2.0) measured with a Nanodrop 2000C spectrophotometer (Thermo Scientific, USA). One microgram of total RNA was used for cDNA synthesis using the TransScript Reverse Transcription Kit (TransGen Biotech, Beijing).

### 4.4 Full-length cDNAs of *BxGST3* and *BxGST1* genes were amplified using RACE technology

Partial sequences of *BxGST3* and *BxGST1*(BXY_1562600, BXY_0594100)were retrieved from the *B. xylophilus* transcriptome data available in the NCBI database (http://www.ncbi.nlm.nih.gov). Primers were designed based on these sequences (**Table 1**). PCR amplification was performed using the reverse−transcribed cDNA as template. After the reaction, 6–10 μL of each PCR product was analyzed by agarose gel electrophoresis (**Supplementary Figure 2**). The target fragments were excised and purified using a gel extraction kit (TaKaRa, Dalian) and subsequently ligated into the pEASY−T1 vector (TransGen Biotech, Beijing). The ligation products were transformed into *Escherichia coli* Trans1−T1 competent cells (TransGen Biotech, Beijing). After transformation, 945 μL of liquid LB medium (without ampicillin) was added to the cells, which were then incubated at 37 °C with shaking at 200 rpm for 1 h. The culture was spread onto selective medium containing ampicillin. Single colonies were picked with a sterile toothpick and inoculated into 500 μL of LB broth supplemented with 100 mg/L ampicillin. The cultures were shaken at 37 °C and 200 rpm for 3–6 h and stored at 4 °C. Colony PCR was used to verify the insertion of the target fragments into the vector. After amplification, 8 μL of each PCR product was examined by 1% agarose gel electrophoresis to confirm successful cloning. Fresh bacterial cultures were sent to GenScript Biotech Co., Ltd. (Nanjing, China) for sequencing. Total RNA was converted into 3’-RACE−ready cDNA and 5’-RACE−ready cDNA using the SMARTer^®^ RACE 5’/3’ Kit (TaKaRa, Dalian). Based on the partial gene sequences obtained above, gene−specific primers for 3’ and 5’ RACE amplification were designed separately (**Table 1**). The 5’-terminal and 3’-terminal sequences of both genes were obtained through 5’-RACE and 3’-RACE PCR reactions (**Supplementary Figure 3**), respectively. Finally, the full−length cDNA sequences of *BxGST3* and *BxGST1* from *B. xylophilus* were assembled by software alignment.

**Table 1:** Primers used in this study.

Name of Primer	Sequence (5'-3')
Cloning of Partial Fragments of *BxGST3* and *BxGST1*	
F-*BxGST*-3	GCTGCTTGCATTATGGTGG
R-*BxGST*-3	ACTTTCGGGAATTGCTTGG
F-*BxGST*-1	CGAATACAGTCTGCACTACTTGAAC
R-*BxGST*-1	GATTGGCAATATATTCTTTCAATTG
cDNA Cloning of *BxGST3* and *BxGST1*	
5'-*BXGST3*-GSP	ATAGGGCCCAGCATCCTTGGTTAGGGCG
5'-*BXGST3*-NGSP	GGAAAGTCTCATCGGCTTTGG
3'-*BXGST3*-GSP	CTCCCAATGGAACCGTGCCGCTTTTCG
3'-*BXGST3*-NGSP	GCCATTCTTCGATACGTATCTAG
5'-*BXGST1*-GSP	CTTCAAATGGGCGAGGAGACAGTCGTGG
5'-*BXGST1*-NGSP	TAATCGGGCAATGTAACGAAGGA
3'-*BXGST1*-GSP	TGAACTGTCGAGGTCTCTGCGAGCCAAC
3'-*BXGST3*-NGSP	AGCCAACAAGGTATTGTCTCCAC
Real time PCR	
*BxGST3*qPCR-F	TGGATACCTCCTAAAATCTGG
*BxGST3*qPCR-R	ATAAACTTTCGGGAATTGCTT
*BxGST1*qPCR-F	CAAGCCTTTGTCTCAATCTGG
*BxGST1*qPCR-R	GAAGAAATGGAAGGTCTCGTC
Actin-F	GCAACACGGAGTTCGTTGTAGA
Actin-R	GTATCGTCACCAACTGGGATGA
Preparation of template DNA for In situ hybridization	
D- *BxGST3*-F	GTTCTAAAGGGTGAATGGG
D- *BxGST3*-R	GCAGTAGTATCAACGCAGAG
D- *BxGST1*-F	TATTCGTTTGCTGAGTTCC
D- *BxGST1*-R	TCCAAGGTGACAATGTTTAT
Preparation of template DNA for dsRNA	
*BxGST3* -F-T7	TAATACGACTCACTATAGGGAGA AAGAGTTCTAAAGGGTGAATGGG
*BxGST3* -R-T7	TAATACGACTCACTATAGGGAGA TCATAAACTTTCGGGAATTGCTT
*BxGST3* -F-T7	TAATACGACTCACTATAGGGAGA TGAGTTCCCAAAGTACAAAGAGT
*BxGST3* -R-T7	TAATACGACTCACTATAGGGAGA AATTGGACAACAAAATCAGCATA
*GFP*-F-T7	TAATACGACTCACTATAGGGAGA CACTTGTCACTACTTTCGGTT
*GFP*-R-T7	TAATACGACTCACTATAGGGAGA GATTCCATTCTTTTGTTTGTCT

The T7 promoter sequences were underlined.

### 4.5 Real-time PCR

qPCR was performed using the AceQ qPCR SYBR Green Master Mix kit (Vazyme, Nanjing) on a QuantStudio 6 Flex system (Applied Biosystems). The cycling conditions were: 95 °C for 5 min; 40 cycles of 95 °C for 15 s and 60 °C for 30 s. Each reaction contained 100 ng of cDNA, 0.2 μM of each primer, and 10 μL of SYBR Green Master Mix in a final volume of 20 μL. The *BxActin* gene was used as the internal reference. Relative expression levels were calculated using the 2^−ΔΔCt method. All reactions were performed in triplicate.

### 4.6 *In situ* hybridization

Specific primers were designed to amplify ~500 bp fragments of *BxGST3* and *BxGST1* (**Supplementary Figure 4**). The *in situ* hybridization method was based on the protocol described by Nielsen et al. (Nielsen & Jones, 2014).The PCR products were labeled with digoxigenin (DIG) using the DIG High Prime DNA Labeling and Detection Starter Kit I (Roche). Hybridization was performed at 42 °C overnight. After washing, color development was carried out according to the manufacturer’s instructions. Nematodes processed without the labeled probe served as the negative control.

### 4.7 RNAi

Target fragments of ~500 bp were selected from the coding sequences of *BxGST3* and *BxGST1* to avoid off-target effects. dsRNA was synthesized using the MEGAscript RNAi Kit (Ambion, USA). dsRNA quality was assessed by 1% agarose gel electrophoresis (**Supplementary Figure 5**) and by OD260/280 ratio(**Supplementary Table S1**) measured with a Nanodrop 2000C spectrophotometer (Thermo Scientific, USA). The dsRNA concentration was adjusted to 800 ng/μL with DEPC-treated water. Nematodes were soaked in dsRNA solution for 48 h at 20-25 °C with gentle shaking (180 rpm) (Liu et al., 2022; Qiu et al., 2016; Xu et al., 2015).

### 4.8 Measurement of reproductive rate in *B. xylophilus* after RNAi

PWNs were separately soaked in ds*BxGST3*, ds*BxGST1*, or ds*GFP* interference solutions, as well as sterile water, and incubated at 20 °C with shaking at 180 rpm for 48 hours. Following the soaking treatment, 15 female and 15 male nematodes from each group were transferred onto Potato Dextrose Agar (PDA) plates fully colonized by *Botrytis cinerea* mycelium. The plates were then incubated at 25 °C in a constant temperature incubator, with three replicate plates set up for each treatment group. Nematode feeding activity was monitored regularly throughout the incubation period and documented with photographs. After 9 days, nematodes were isolated and collected. The number of nematodes for each treatment was counted under a microscope to calculate the reproduction multiplier. The counting procedure involved pipetting 50 µL of a thoroughly mixed nematode suspension; this sampling and counting process was repeated 5 to 10 times, and the average value was used for the final calculation.

### 4.9 Analysis of pathogenicity in *B. xylophilus* following RNAi

*B. xylophilus* nematodes previously soaked in ds*BxGST3*, ds*BxGST1*, ds*GFP* interference solutions, or sterile water were inoculated separately onto two-year-old *Pinus thunbergii* seedlings using the bark-inoculation method. For each seedling, approximately 10 cm above the ground, 1500 nematodes (10 nematodes/μL, 150 μL) were inoculated. Seedlings inoculated with 150 μL of sterile water alone served as controls. Two replicates were performed for each treatment. Control 1: 150 μL of deionized water; Control 2: 150 μL of nematodes soaked in sterile water; Control 3: 150 μL of nematodes soaked in ds*GFP* interference solution; Treatment 1: 150 μL of nematodes soaked in ds*BxGST3* interference solution; Treatment 2: 150 μL of nematodes soaked in ds*BxGST1* interference solution. After inoculation, disease symptoms on the *P. thunbergii* seedlings were monitored regularly and photographed.

## Data Availability

The datasets presented in this study can be found in online repositories. The names of the repository/repositories and accession number(s) can be found in the article/**Supplementary Material**. Sequences have been deposited in GenBank under accession numbers PZ190912 and PZ190913.

## Supplementary material

Supplementary Figure 1 Total RNA from *B.xylophilus.* (Lane 1, DNA Marker; Lane 2, RNA bands).

Supplementary Figure 2 Electrophoretic analysis of partial fragments of *BxGST3* and *BxGST1* amplified from *Bursaphelenchus xylophilus* genomic cDNA. (Lane 1, DNA marker; Lane 2, *BxGST3*; Lane 3, *BxGST1*).

Supplementary Figure 3 Analysis of 3’-RACE and 5’-RACE electrophoretic profiles of *BxGST3* and *BxGST1* from *Bursaphelenchus xylophilus*. (Lane 1, DNA marker; Lane 2, *BxGST3* 5’-RACE; Lane 3, *BxGST3* 3’-RACE Lane 4, DNA marker; Lane 5, *BxGST1* 5’-RACE; Lane 6, *BxGST1* 3’-RACE).

Supplementary Figure 4 Electrophoretic analysis of digoxigenin (DIG)-labeled gene fragments of *BxGST3* and *BxGST1*. (Lane 1, DNA marker; Lane 2, *BxGST3*; Lane 3, *BxGST1*).

Supplementary Figure 5 Agarose gel electrophoresis of dsRNA for genes of *BxGST3*、*BxGST1*、*GFP*. (Lane 1, DNA marker; Lane 2–4, ds*BxGST3*; Lane 5, 6, ds*BxGST1* Lane 7, DNA marker; Lane 8, 9, ds*GFP*. Three replicate amplification experiments were performed for each group, and only the lanes with successful amplification are labeled in the figure).
