# Supplementary figures and images for: Cloning and functional analysis of glutathione S-transferase gene BxGST3 and BxGST1 in Bursaphelenchus xylophilus
Source: Front Plant Sci. 2026 May 25;17:1847982. doi: 10.3389/fpls.2026.1847982 (PMC13243375; doi:10.3389/fpls.2026.1847982)

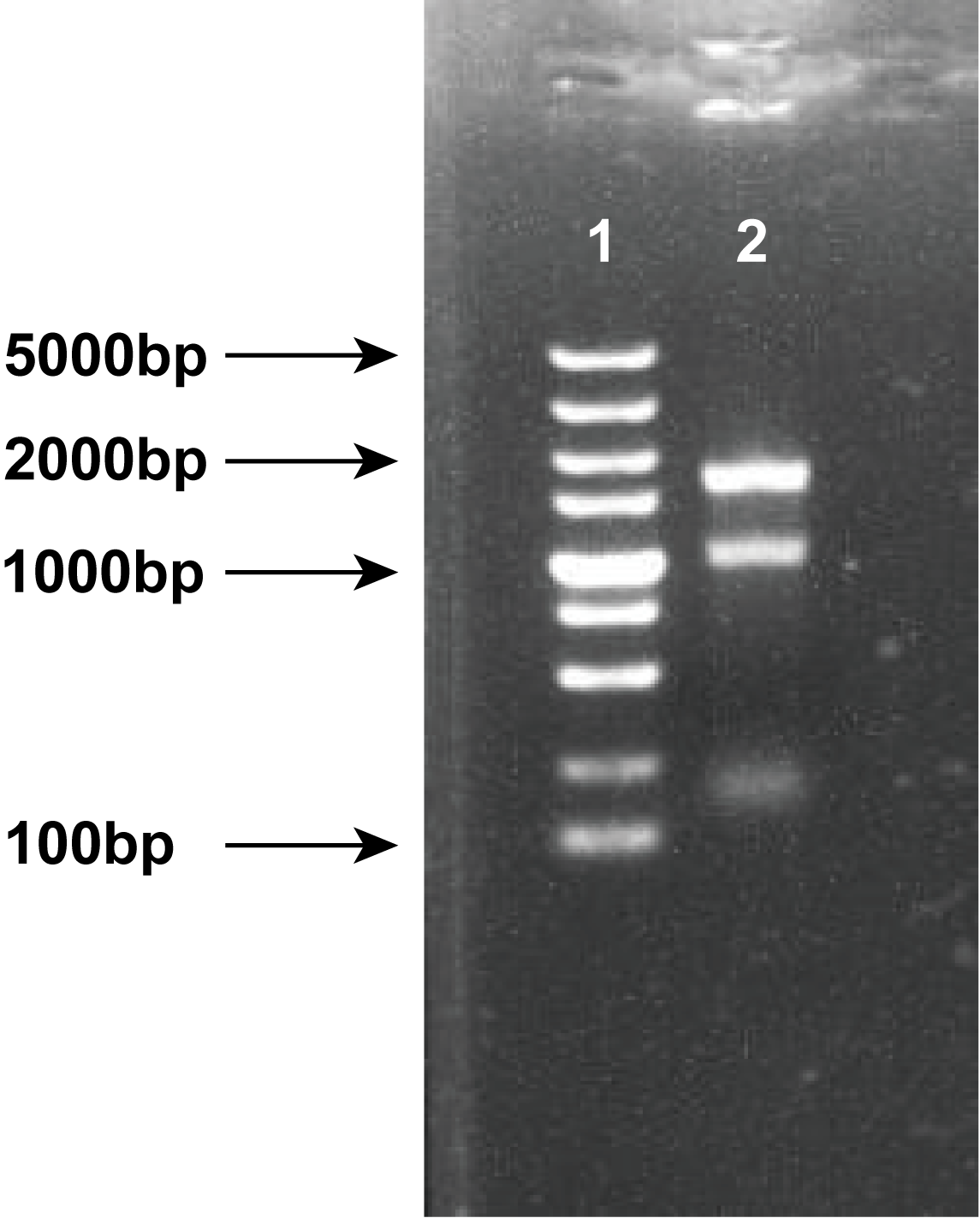

Supplement: Supplementary Figure 1 — Total RNA from B.xylophilus. (Lane 1, DNA Marker; Lane 2, RNA bands). [file Image1.tif]

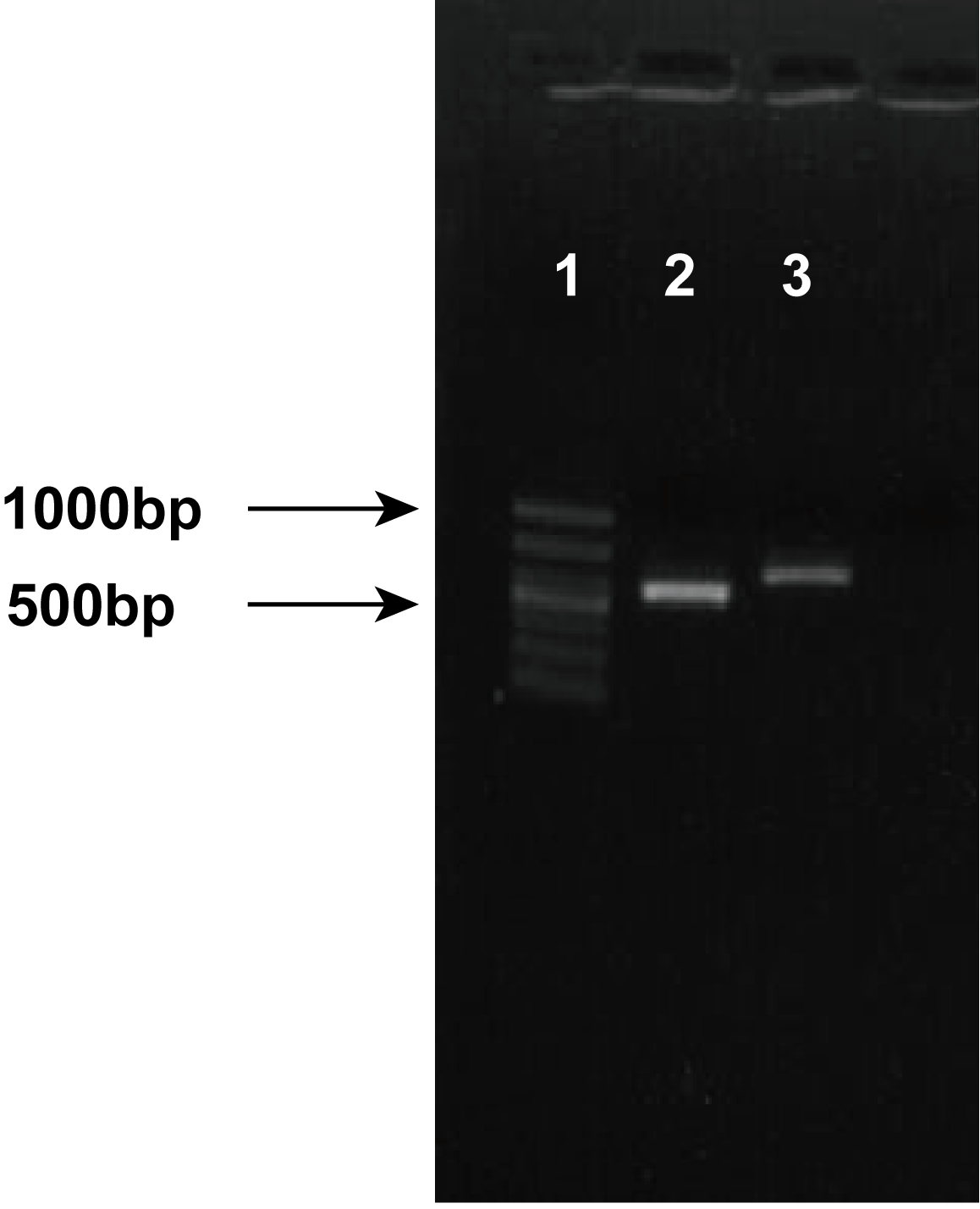

Supplement: Supplementary Figure 2 — Electrophoretic analysis of partial fragments of BxGST3 and BxGST1 amplified from Bursaphelenchus xylophilus genomic cDNA. (Lane 1, DNA marker; Lane 2, BxGST3; Lane 3, BxGST1). [file Image2.tif]

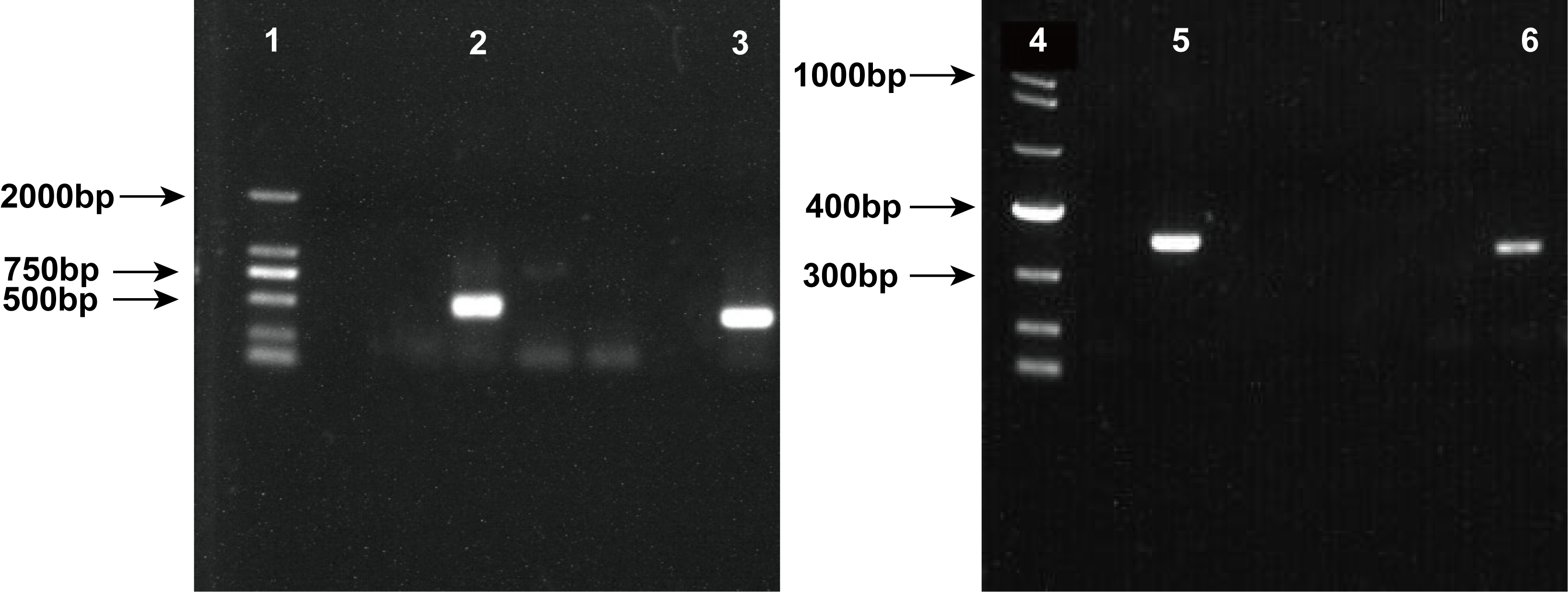

Supplement: Supplementary Figure 3 — Analysis of 3’-RACE and 5’-RACE electrophoretic profiles of BxGST3 and BxGST1 from Bursaphelenchus xylophilus. (Lane 1, DNA marker; Lane 2, BxGST3 5’-RACE; Lane 3, BxGST3 3’-RACE Lane 4, DNA marker; Lane 5, BxGST1 5’-RACE; Lane 6, BxGST1 3’-RACE). [file Image3.tif]

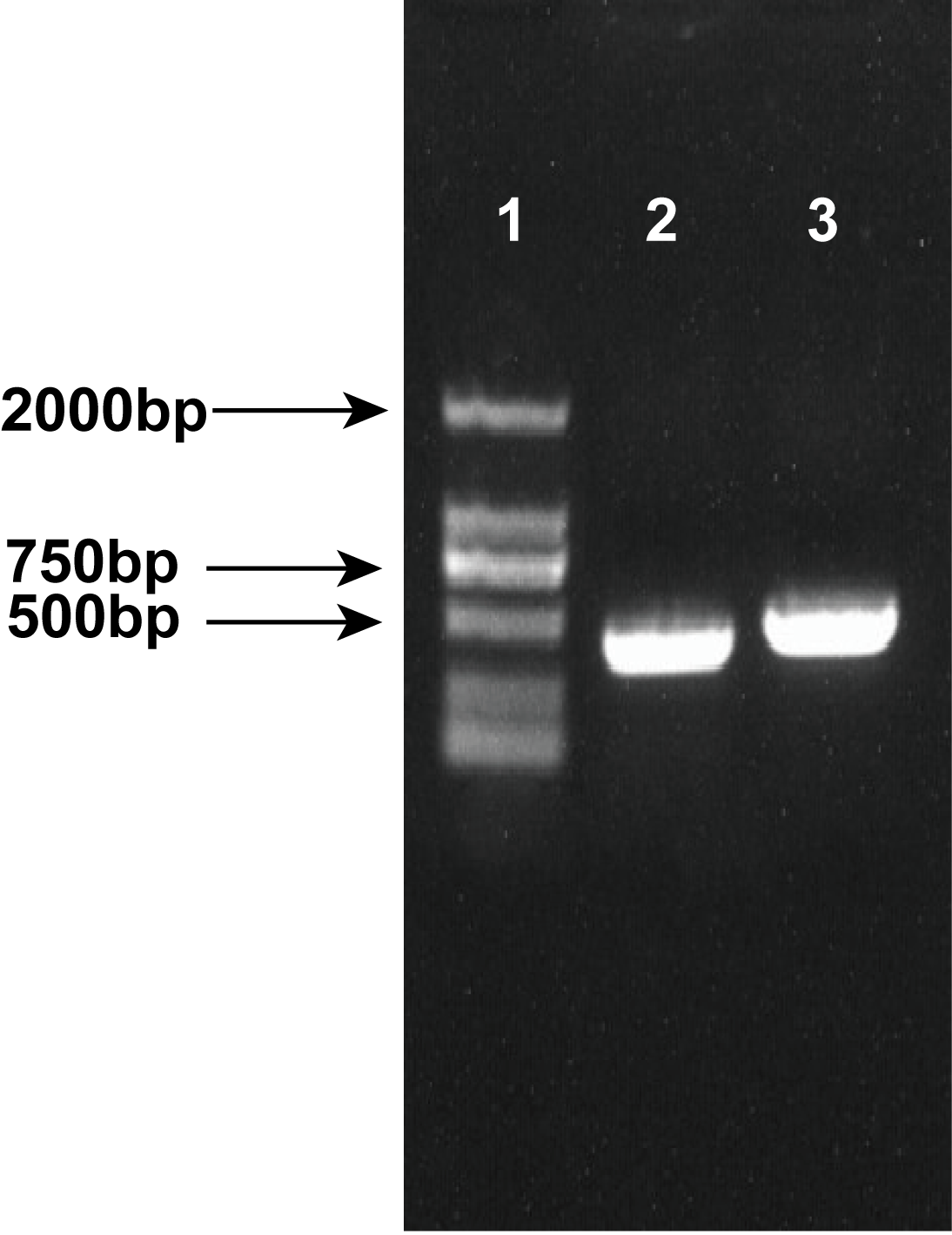

Supplement: Supplementary Figure 4 — Electrophoretic analysis of digoxigenin (DIG)-labeled gene fragments of BxGST3 and BxGST1. (Lane 1, DNA marker; Lane 2, BxGST3; Lane 3, BxGST1). [file Image4.tif]

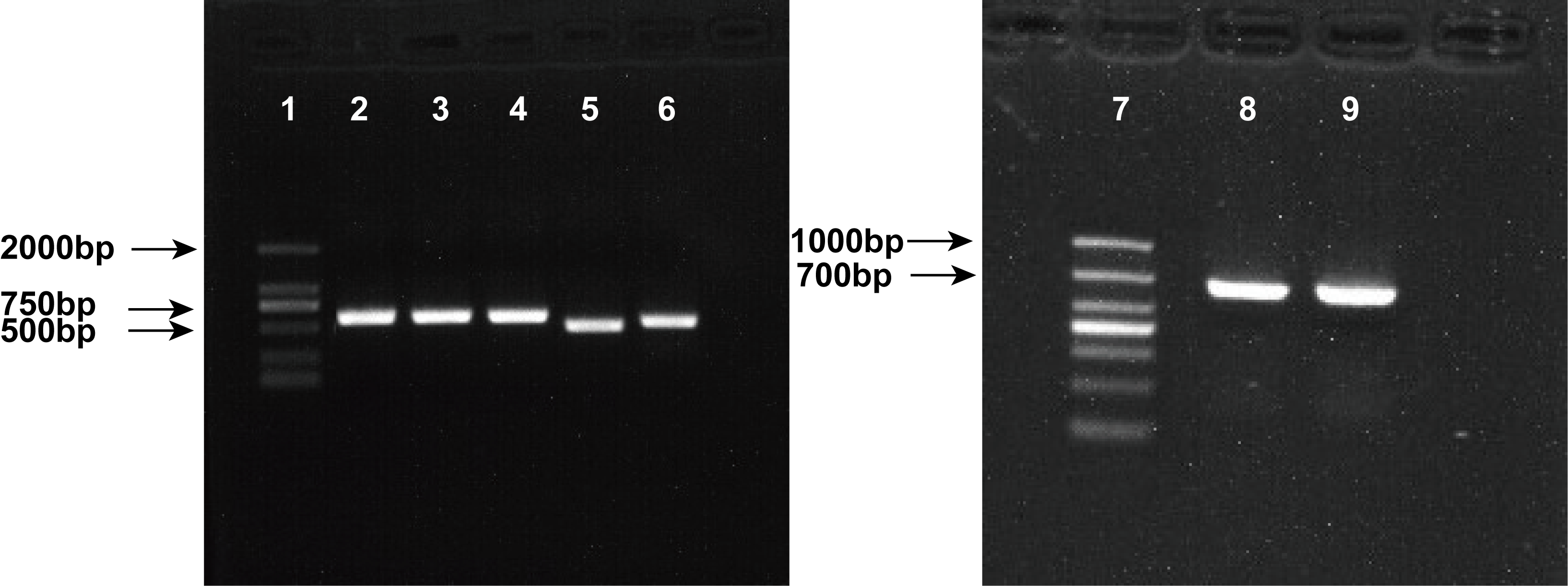

Supplement: Supplementary Figure 5 — Agarose gel electrophoresis of dsRNA for genes of BxGST3、BxGST1、GFP. (Lane 1, DNA marker; Lane 2–4, dsBxGST3; Lane 5, 6, dsBxGST1 Lane 7, DNA marker; Lane 8, 9, dsGFP. Three replicate amplification experiments were performed for each group, and only the lanes with successful amplification are labeled in the figure). [file Image5.tif]
