# Supplementary material for: Cloning and functional analysis of glutathione S-transferase gene BxGST3 and BxGST1 in Bursaphelenchus xylophilus
Source: Front Plant Sci. 2026 May 25;17:1847982. doi: 10.3389/fpls.2026.1847982 (PMC13243375; doi:10.3389/fpls.2026.1847982)
Supplement: Supplementary file 6 [file Table1.docx]

Table S1 The result of ultraviolet spectrophotometer of dsRNA of genes for *BxGST3*、*BxGST1*、*GFP*

| Sample Name | A260/A280 | Concentration (μg/μL) | Volume（μL） | Total（μg） |
| --- | --- | --- | --- | --- |
| *BxGST3*① | 1.98 | 1.666 | 30 | 4.998 |
| *BxGST3*② | 1.94 | 1.285 | 30 | 3.855 |
| *BxGST3*③ | 1.95 | 1.376 | 30 | 4.128 |
| *BxGST1*① | 2.01 | 0.953 | 30 | 2.859 |
| *BxGST1*② | 1.91 | 1.219 | 30 | 3.657 |
| *GFP*① | 2.02 | 1.399 | 30 | 4.197 |
| *GFP*② | 1.89 | 1.976 | 30 | 5.928 |
